# Synthesis, Crystal Structure, and Transport in Ordered Vacancy Compound Hg_2_SiTe_4_

**DOI:** 10.1021/acs.inorgchem.5c05605

**Published:** 2026-03-30

**Authors:** Claire E. Porter, Jiaxing Qu, Philip Yox, Terra Berriodi, Annalise E. Maughan, Elif Ertekin, Eric S. Toberer

**Affiliations:** † Materials Science, 3557 Colorado School of Mines, Golden, Colorado 80401, United States; ‡ Mechanical Science & Engineering, 14589 University of Illinois at Urbana−Champaign, Urbana, Illinois 61801, United States; § Chemistry, Colorado School of Mines, Golden, Colorado 80401, United States

## Abstract

Hg_2_SiTe_4_ is found to be stable at room temperature and pressure and crystallizes in the *I*4̅ space group. The tetragonal compound (*a* = 6.04681(3) Å, *c* = 12.01208(9) Å, *Z* = 2) exhibits *p*-type electronic conduction and ultralow thermal conductivity (κ_total_ < 1 W/m K at 50 °C). The crystal structure is determined via charge flipping using TOPAS software to analyze X-ray diffraction data on powder samples. The compound is synthesized from elemental precursors and requires densification before annealing to form the ternary compound, unlike the similar compound Hg_2_GeTe_4_. Several synthetic procedures are tested to determine the best method to form the novel compound. Measured thermal and electronic property data are discussed along with first-principles defect calculations to inform future doping or optimization studies. Defect calculations suggest that ${\mathrm{Si}}_{\mathrm{Hg}}^{2+}$ , $V_{\mathrm{Hg}}^{2-}$ , and ${\mathrm{Hg}}_{\mathrm{Si}}^{2-}$ native antisite defects may pin the Fermi level deep within the bandgap. Throughout, we compare the crystallographic and electronic properties of Hg_2_SiTe_4_ to those of the similar, previously discovered compound Hg_2_GeTe_4_.

## Graphical abstract

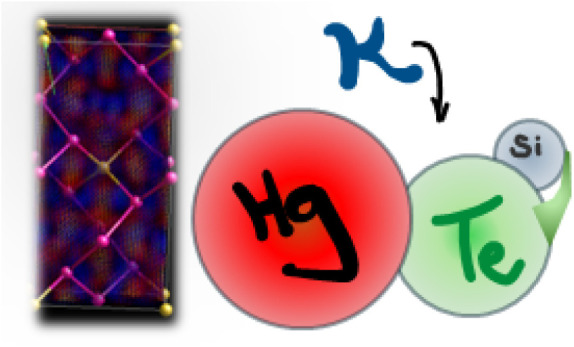

## Introduction

In the context of thermoelectric materials, ultralow thermal conductivity is desirable while retaining electronic conductivity. In general, diamond-like semiconductors are considered to be poor thermoelectric materials due to their strong covalent bonding, which leads to high thermal conductivity. For example, SiGe alloys, which have been pursued by NASA for decades as a thermoelectric material, must incur significant particle size engineering to scatter phonons and thereby reduce thermal conductivity and render them useful as thermoelectric materials.^1–3^ Hg-containing diamond-like semiconductors have been identified previously for their anomalously low thermal conductivity.^4–6^ The relatively large Hg atoms lead to softer bonding and enhanced scattering of phonons, thereby effectively limiting thermal conduction.^4^ High-throughput searches for the next excellent thermoelectric material have yielded several yet-unsynthesized compounds in the ternary Hg-containing chalcogenide space.^7^

The group of *A*_2_ *BX*_4_ materials, with metallic *A* and *B* elements and *X* as a chalcogen, are important across various domains, including nonlinear optics, photovoltaic power generation, and thermoelectric materials.^4,6–8^ Their technological importance has spurred several computational forays into exploring the thermodynamic stability of new phases, which offer potentially game-changing properties. For example, a 2012 computational survey of phase stability across the *A*_2_ *BX*_4_ material space identified 100 compounds predicted to be thermodynamically stable out of the 429 unreported compounds where *A* and *B* are main group elements and *X* is O, S, Se, or Te. One of these unreported yet predicted stable compounds was Hg_2_SiTe_4_, warranting an experimental study to determine its phase stability. Known stable ternary phases of Hg-containing chalcogenides include the selenides Hg_2_GeSe_4_ and Hg_2_SnSe_4_ as well as the oxide Hg_2_GeO_4_.^7^ Of note, Hg_2_GeTe_4_ was predicted in this study to not form a stable ternary compound, but it was successfully synthesized years later.^5^ Meanwhile, Hg_2_SiTe_4_ is predicted to be stable.^7^ Successful synthesis of Hg_2_Si*X*_4_ is unreported to date across the chalcogen space (*X* = O, S, Se, or Te). Considering the successful synthesis of Hg_2_GeTe_4_ and the ultralow thermal conductivity of this family of Hg-containing chalcogenides, we pursue our investigation of Hg_2_SiTe_4_.

In this study, we report our successful synthesis of Hg_2_SiTe_4_, along with detailed crystallographic information, calculated native defect formation energies, measured transport properties, and observed thermal stability of the compound. Ultimately, we successfully resolve the structure of the compound and confirm its ultralow thermal conductivity. While the thermal conductivity is quite promising for thermoelectric applications, the compound suffers from low electronic mobility and conductivity that could be improved with doping.

## Experimental Section

### Synthesis

**Caution!** Mercury and tellurium are toxic if inhaled, and ball milling can generate airborne powder. All handling was performed in an inert N_2_-filled glovebox.

Samples were prepared from elemental precursors of high purity (Hg, liquid, Alfa 99.999%, Si, ingot; Te, ingot, 5NPlus Inc., 99.999%) under solid-state reaction methods. Elements were weighed in an inert N_2_ glovebox containing less than 1 ppm of O_2_ to yield about 10 g total of the stoichiometric ratios Hg_2_SiTe_4_. The weighed elements were loaded into a tungsten carbide ball mill vial with two balls in the glovebox and milled for 15 min to mix the elements and react liquid Hg to form a solid, dark gray powder, with no liquid phase present. The resulting powder was loaded into a graphite die and subjected to a 15 min uniaxial press at 40 MPa with no heat applied in an evacuated chamber. The formed pellet was returned to the inert glovebox, where it was hand-ground with an agate mortar and pestle and loaded into a clean quartz ampule. The quartz ampule was evacuated and sealed using a vacuum pump and torch, and the ingot was annealed at 400 °C for 72 h and allowed to cool slowly to promote the sample achieving equilibrium. Cooled ampules were broken open, the ingot was extracted, and the powder was hand-ground and passed through a 200 mesh sieve, all inside the inert glovebox.

Annealed powder as described above was used for pXRD experiments (Figure 1), and densified pellets were used for microscopy experiments (Figure 2), electronic measurements (Figure 3), and determination of thermal conductivity (Figure 4a). To form pellets, 3 g of the powder prepared as described above were loaded into a graphite die and hot-pressed under vacuum at 400 °C for at least 6 h to form a consolidated pellet unless stated otherwise. Pellets were polished to a flatness of ±5 μm for measurements.

**Figure 1:**
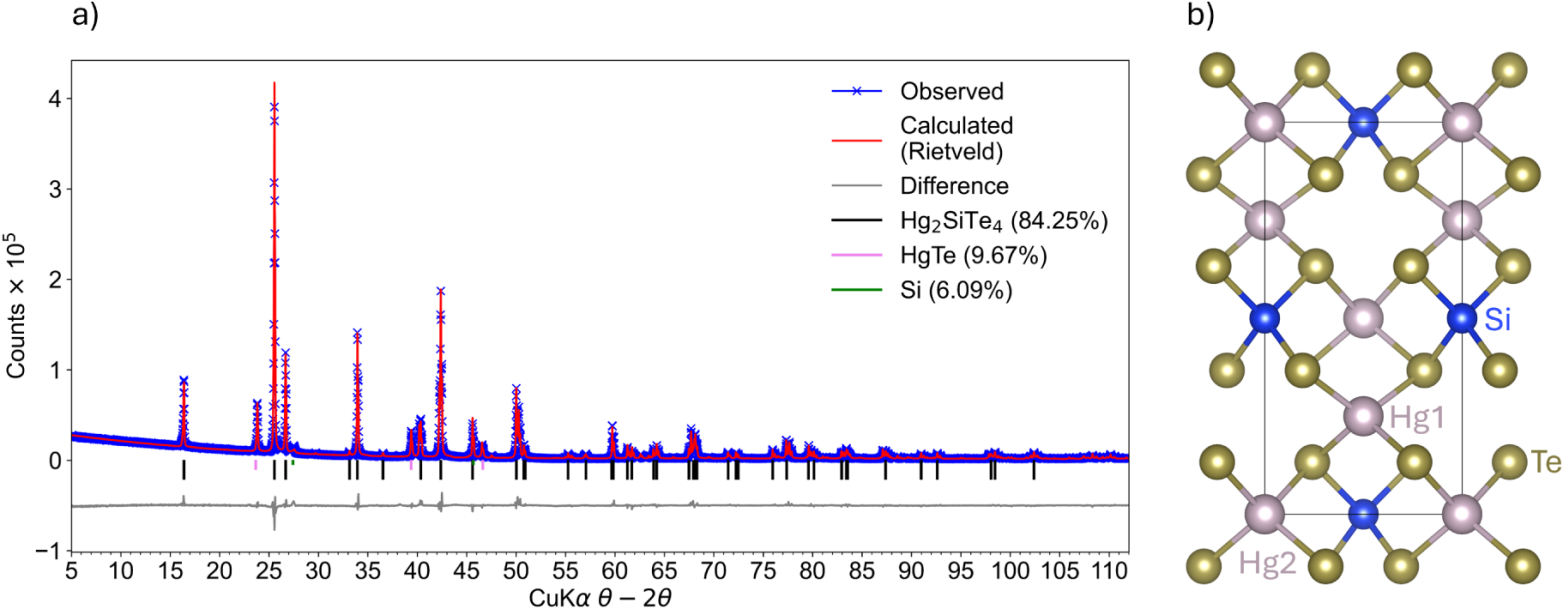
(a) Rietveld refinement of the *I*4̅ structure against laboratory powder X-ray diffraction data for Hg_2_SiTe_4_ containing some impurity phases. (b) The *I*4̅ structure contains ordered vacancies within the unit cell. This is the same crystallographic structure adopted by Hg_2_GeTe_4_.

**Figure 2:**
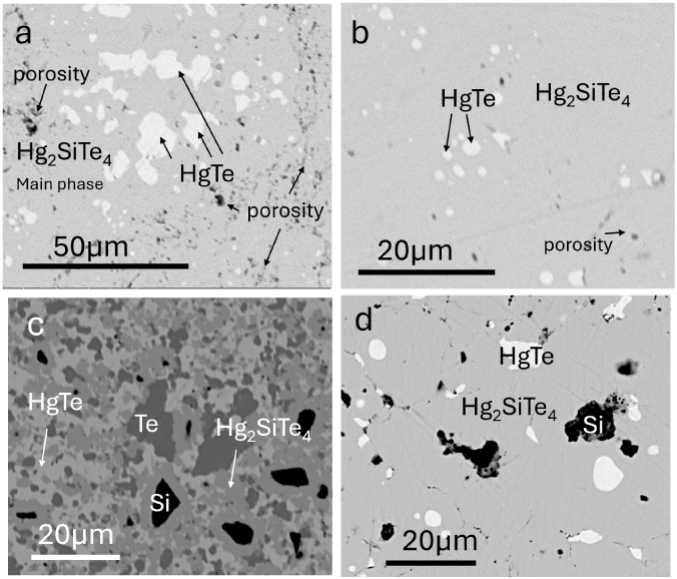
Different synthetic conditions lead to different impurity phases, as shown in the SEM images. Samples hot-pressed at 400 °C (a,b,d) show a main phase of Hg_2_SiTe_4_. The sample in (a) was used for electronic transport property measurements (Figure 3). The sample pressed at 350 C (c) contains large inclusions of unreacted elemental Te and Si.

**Figure 3:**
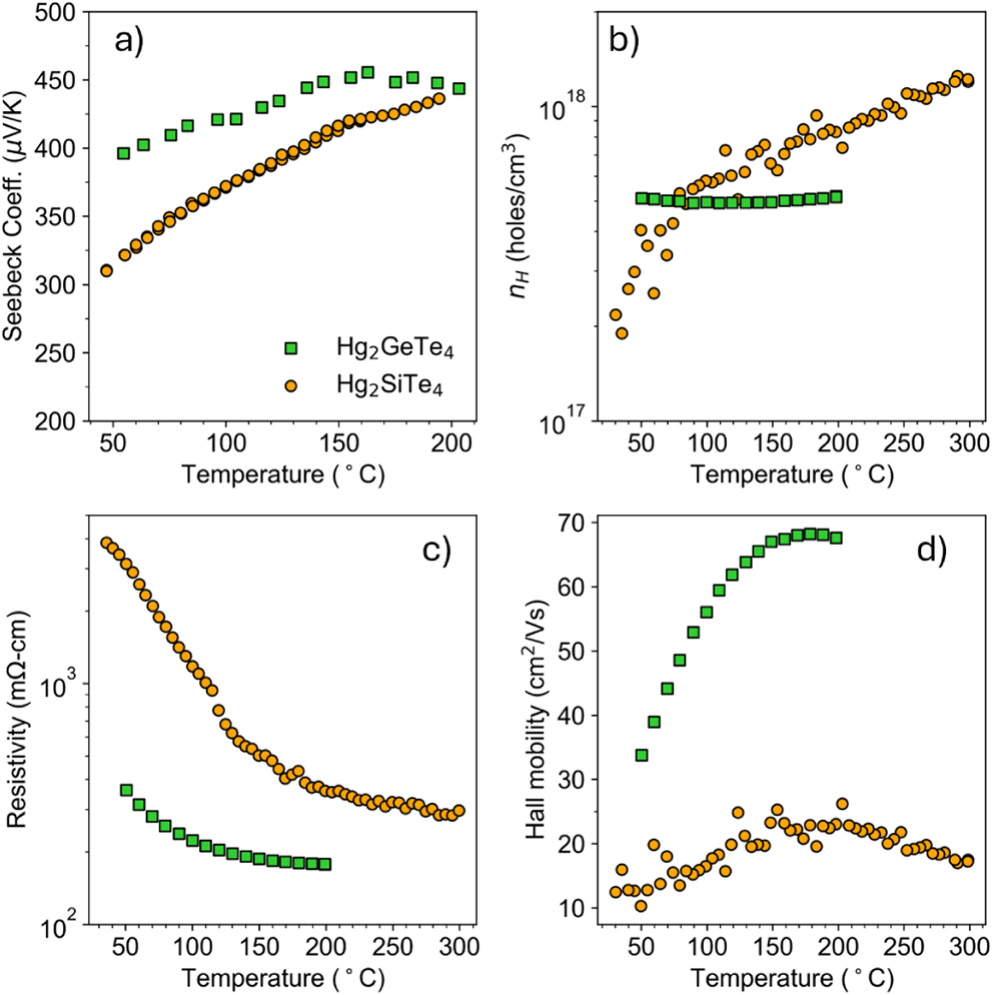
Hg_2_SiTe_4_ (orange circles) demonstrates *p*-type intrinsic semiconductor behavior. Electronic transport data for the relatively more conductive Hg_2_GeTe_4_ (green squares) are shown alongside for comparison. Data for Hg_2_GeTe_4_ are reproduced from ref 6.

**Figure 4:**
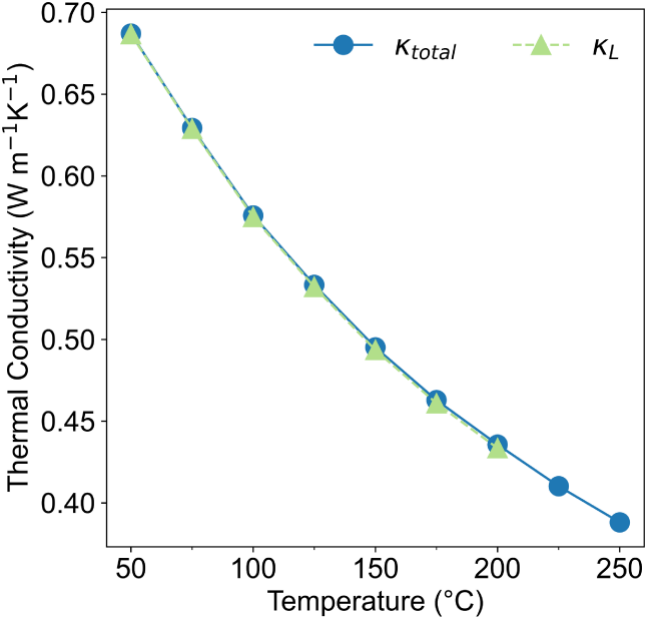
Lattice thermal conductivity accounts for over 99% of the total thermal conductivity in a polycrystalline sample of Hg_2_SiTe_4_.

### Powder X-ray Diffraction

Powder X-ray diffraction (pXRD) experiments were performed using a Panalytical Empyrean X-ray diffractometer at room temperature and ambient pressure in Golden, Colorado, using a Cu Kα radiation source (λ = 1.5406 Å). Bragg–Brentano geometry was employed with a scan range of 2θ = 5–120° with a scan step size of 2θ = 0.014324° for a total of 8029 points. The total scan time was approximately 1 h. The generator voltage was 45 kV, and the tube current was 40 mA. The structure of Hg_2_SiTe_4_ was solved from this pXRD (Figure 1), and the crystallographic and Rietveld refinement results are tabulated in Table 1.

**Table 1:** Crystallographic Data Obtained from Rietveld Refinement of the Powder (Figure 1)

Hg_2_SiTe_4_	939.67 g/mol
*a* = 6.04681(3) Å	space group *I*4̅ (No. 82)
*b* = 6.04681(3) Å	*T* = 293 K, ambient pressure
*c* = 12.01208(9) Å	λ = 1.540598 Å
*V* = 439.208(6) Å^3^	*D*_*calc*_ = 7.10530(9) g/cm^3^
*Z* = 2	*R*_*w*_ = 7.00, *R*_*exp*_ = 3.54, χ^2^ = 1.98

Other laboratory pXRD experiments were performed using a Bruker D6 Phaser at a faster scan rate and a lower 2θ range to test the formation of the ternary compound during various synthetic routes tested. Results from these pXRD experiments as well as photos of the experiments, are provided in the Supporting Information (Figures S1–S7).

### Structure Solving

To solve the crystallographic structure of Hg_2_SiTe_4_, we performed a charge-flipping routine^9^ using TOPAS Academic v6.^10^ To generate possible space groups for this structure, we performed an iterative loop of peak indexing, Pawley refinement, charge flipping, and Rietveld calculations until we realized the best fit. All of these steps were performed by using the TOPAS software. We found that the best-fit space group is *I*4̅, which is the same structure as Hg_2_GeTe_4_.^5^

### Scanning Electron Microscopy and Energy Dispersive Spectroscopy

We conducted scanning electron microscopy (SEM) experiments and elemental mapping using energy dispersive spectroscopy (EDS) with an FEI Quanta 600i SEM. Pressed pellets polished to a mirror finish using typical techniques were used for this study. EDS was performed at 20 kV, and the HgL, SiK, and TeL transition levels were considered. In Figure 2, we highlight samples of varying purity to demonstrate the impact of anneal temperature on phase purity.

### Electronic Transport Measurements

Hall effect, electrical resistivity, and Seebeck coefficient measurements were performed on custom apparatuses^11,12^ from room temperature up to 200 °C for Seebeck, and 300 °C for resistivity and Hall measurements. The specimen measured was a bulk, polycrystalline, undoped, polished pellet of Hg_2_SiTe_4_ (Figure 2d). Carrier concentration was calculated assuming a single parabolic band model, *n* = *r*_*H*_/*eR*_*H*_, where *r*_*H*_ was set to equal unity. Hall mobility μ was calculated from the Drude equation (σ = *n*_*H*_ *e*μ).

### Thermal Measurements

Thermal diffusivity (*D*) was measured by a laser flash method (LFA467, Netzsch). Total thermal conductivity (κ) was calculated from the following equation: κ = *DC*_*p*_ *d*, where *d* is the sample density measured geometrically from a cylindrical pellet with flatness ±10 μm (*d* = 6.856 g/cm^3^), and *C*_*p*_ was determined by the Dulong–Petit law. In addition to thermal diffusivity, we performed differential scanning calorimetry (DSC) experiments in an effort to determine the melting point of the compound as well as the temperature of the reaction to form the ternary.

## Computational Section

### Density Functional Theory (DFT) Calculations

The first-principles density functional theory (DFT) calculations were performed using the plane-wave basis Vienna *Ab initio* Simulation Package.^13^ The Perdew–Burke–Ernzerhof (PBE)^14^ exchange–correlation functional was used within the generalized gradient approximation. We adopted the projector-augmented wave^15^ pseudopotentials to represent core and valence electrons. The hypothetical structures generated by using chemical substitutions were relaxed with a plane-wave energy cutoff of 340 eV and an automatically generated Γ-centered regular *k*-point mesh to sample the Brillouin zone for the primitive cells. During structural relaxation, the convergence criteria for energy and force relaxations are set as 10^–6^ eV and 10^–5^ eV Å^–1^, respectively. The relaxed crystal structure was then used consistently for the following defect calculations. The electronic structures were calculated from the charge density of the fully relaxed structure using the tetrahedron method for *k*-point integration and a dense *k*-mesh with a fixed number of *k*-points of 8000 per unit cell. To calculate defect energetics, we used the standard supercell approach^16^ to determine the defect formation energies of native point defects.

## Results and Discussion

### Synthesis and Phase Stability

Synthesis of a phase-pure sample of Hg_2_SiTe_4_ was difficult. While HgTe forms readily from ball milling alone, and Hg_2_GeTe_4_ requires an anneal at 350 °C post-ball milling to form,^4,6,17^ we have found that Hg_2_SiTe_4_ requires ball milling and densification prior to annealing to form the ternary compound. This is likely because solid-state diffusion is the limiting step of the reaction to form the ternary species, especially considering the large atomic radii difference across the chemical species.

Ultimately, we found that Hg_2_SiTe_4_ adopts the same *I*4̅ structure as the related compound Hg_2_GeTe_4_ that can be described as two stacked zinc blende cells containing ordered vacancies. The phase purity of the sample is 84.25 wt % Hg_2_SiTe_4_ from XRD (Figure 1), which we acknowledge is lower in purity than some of our other syntheses (for example, Figure 2b is almost pure polycrystalline Hg_2_SiTe_4_). We chose to perform our refinement on this sample because, at that time, we had access to an X-ray diffractometer with a far superior signal-to-noise ratio, and we were able to distinguish the peaks of impurity phases Si and HgTe from the main matrix phase Hg_2_SiTe_4_.

The synthetic difficulty we experienced in realizing high-phase-purity Hg_2_SiTe_4_ can be largely attributed to its limited phase stability. Using first-principles calculations, we calculated the energy of the phase in the *I*4̅ structure to be 0 eV above the convex hull *E*_hull_, suggesting a stable compound. However, competing phases—HgTe, Te, and Si—limit the range of chemical potential values that Hg_2_SiTe_4_ can adopt (Table 2). Under the most Hg-poor (Si-rich/Te-rich) conditions, elemental Si and Te limit the chemical potential of the ternary complex (middle row of Table 2). When the Hg chemical potential is maximized (bottom row of Table 2), the competing phases formed are Si and HgTe. Values of Δμ_i_ for all synthetic conditions are provided in Table 2 below.

**Table 2:** Chemical Potentials Δ*μ*_i_ (in eV) across the Phase Stability Region for Hg_2_SiTe_4_ ^a^

Compound	Equilibrium Phases	Δμ_Hg_	Δμ_Si_	Δμ_Te_
Hg_2_SiTe_4_	Te, HgTe	–0.278	–0.055	0
	Si, Te	–0.306	0	0
	Si, HgTe	–0.250	0	–0.028

a The elemental reference chemical potentials $\mu_{i}^{0}$ are −0.452, −5.57, and −3.022 eV for Hg, Si, and Te respectively.

SEM experiments revealed that HgTe is our most prevalent impurity phase (Figure 2a–d). Elemental mapping using energy dispersive spectroscopy (EDS) was performed to verify the composition of each phase, and at least five points per phase were measured per sample. Low standard deviations in atomic % (at. %) across each phase suggest good homogeneity within each phase labeled in Figure 2. For example, we collected EDS data at 10 different locations far from each other in the main phase (Hg_2_SiTe_4_) for the sample shown in Figure 2d and found excellent homogeneity. We found an average at. % of: 16.71 at. % Si (std dev 1.19 at. %), 27.97 at. % Hg (std dev 0.37 at. %), and 55.32 at. % Te (std dev 0.97 at. %). Normalized to Te, the stoichiometry from EDS is Hg_2.0_Si_1.2_Te_4.0_. We note the semiquantitative nature of EDS. In an effort to eliminate the impurity HgTe, we synthesized samples slightly Si-rich, but this did not resolve the impurity.

Interestingly, the microscopy images in Figure 2a–c correspond to different samples of nominally stoichiometric Hg_2_SiTe_4_. While (a) and (b) are very homogeneous and exhibit over 80% Hg_2_SiTe_4_, (c) is clearly not phase-pure. The explanation for this drastic contrast in phase purity lies in the synthetic conditions: (c) was hot-pressed at 350 °C, whereas (a–b) were hot-pressed at 400 °C. This result demonstrates the importance of a 400 °C anneal to form Hg_2_SiTe_4_. Small inclusions of HgTe in (a–b), despite careful weighing, support our finding above that the predicted phase stability region is limited for this material.

Further discussion of the four-phase sample in Figure 2c is warranted. Notably, the existence of four phases in a ternary system defies Gibb’s phase rule and therefore suggests that this sample has not achieved thermodynamic equilibrium. It is possible that the reaction pathway to synthesize the ternary is diffusion-limited. Interestingly, all instances of elemental silicon (black regions in SEM images; Figure 2c) are encased in a shell of Hg_2_SiTe_4_. Elemental Te, on the other hand, exists without such a shell. The Si/Hg_2_SiTe_4_ clusters exist in a matrix of HgTe, which could suggest that the reaction pathway for forming the ternary compound involves first forming HgTe and then incorporating Si and excess Te. This topotactic-like mechanism is supported by the fact that HgTe adopts a similar structure type to the final ternary product. Incorporating Si may be the rate-limiting step of this reaction, which could be explained by the large contrast in atomic radii between Hg and Si.

### Electronic Measurements

To explore the electronic properties of Hg_2_SiTe_4_, we perform room to high-temperature Hall effect, electrical resistivity, and Seebeck coefficient measurements on a high-purity sample of Hg_2_SiTe_4_. We observe positive Seebeck and Hall coefficient data, suggesting *p*-type conduction for this compound (Figure 3). Data for Hg_2_GeTe_4_ synthesized using similar methods (ball milling, annealing, hot pressing) are shown in Figure 3 for comparison (data adapted from ref 6). Hg_2_GeTe_4_ experiences a rollover in the temperature-dependent Seebeck coefficient around 150 °C, demonstrating the onset of minority carriers. From the maximum Seebeck value, we can calculate the Goldsmid–Sharp band gap (*E*_g_ = 2*e*|*S*_max_| *T*_max_) as 0.40 eV. However, the silicon counterpart does not show evidence of a rollover (Figure 3a) and might melt before this rollover is observed. This, along with resistivity an order of magnitude higher than the Ge analogue with a strong negative temperature dependence, suggests very intrinsic semiconductor behavior and a wide bandgap for Hg_2_SiTe_4_.

Hg_2_SiTe_4_ possesses lower Seebeck coefficient and lower carrier concentration (*n*_*H*_) than the Ge analog at room temperature (Figure 3a,b). This is not beneficial to thermoelectric performance. However, at elevated temperatures, the performance improves and approaches that of Hg_2_GeTe_4_. The resistivity of Hg_2_SiTe_4_ is elevated above that of the Ge analog across all temperatures (Figure 3d), which at first glance seems counterintuitive since its Seebeck coefficient is lower. However, this can, in part, be explained by the lower mobility in the Si compound versus the Ge compound (Figure 3d). This trend was observed in the copper-containing quaternary analogs as well: Cu_2_Hg**Ge**Te_4_ possesses higher mobility than Cu_2_Hg**Si**Te_4_.^4^

Finally, we note that the Hall mobility is very low and exhibits a positive temperature dependence (Figure 3d), suggesting that ionized impurity scattering is the dominant scattering mechanism. The low measured carrier concentration (10^17^ to 10^18^ *h* ^+^/cm^3^) weakens screening, and carriers are easily scattered by charged defects. Our defect calculations provide evidence for an abundance of charged native defects, which is discussed in a subsequent section. Additionally, the low mobility can also be attributed to the large effective mass of the valence band, which is typical of ordered vacancy chalcogenides.^5,6^ Finally, the large mass contrast and potential fluctuation disorder due to the mixing of heavy Hg and light Si atoms impact scattering and also contribute to the ultralow measured thermal conductivity.

### Thermal Analysis

We expected low thermal conductivity in this compound due to prior literature predictions in covalently bonded chalcopyrite compounds containing Te and Hg.^4^ For example, Hg_2_GeTe_4_ is demonstrated to possess ultra-low thermal conductivity, with undoped material possessing a κ_*L*_ of 1.0 W m^–1^K^–1^ at 50 °C.^5,6^ Using the flash diffusivity technique described in the Experimental Section, we calculated the total thermal conductivity of Hg_2_SiTe_4_ to be 0.69 W m^–1^ K^–1^ at 50 °C (Figure 4a), which is even lower than that of Hg_2_GeTe_4_. We note that our sample was not completely phase-pure; the sample measured corresponds to that shown in Figure 2d which contains impurity phases Si and HgTe. However, since Si and HgTe have substantially higher thermal conductivities than 1 W m^–1^ K^–1^, our reported thermal conductivity likely suggests a high-end estimate for the actual thermal conductivity of pure Hg_2_SiTe_4_. To model the true thermal conductivity of pure Hg_2_SiTe_4_, we apply an effective medium theory approach^18^ and factor in the contribution from Si or HgTe. We consider 90 vol% matrix phase Hg_2_SiTe_4_ and then perform two separate calculations considering 10 vol% silicon or HgTe. We calculate the room temperature κ_*T*_ of pure Hg_2_SiTe_4_ to be between 0.49 and 0.58 W m^–1^ K^–1^ using this approach. The lower end of this range corresponds to silicon as the impurity phase (κ at 300 K = 142 W m^–1^ K^–1^)^19^ and the upper end considers HgTe (κ at 300 K = 2.7 W m^–1^ K^–1^).^20^ We justify our use of κ at 300 K (we only measure down to 323 K) for the impurity phases by the fact that $\frac{d\kappa }{dT}$ is not high enough over this temperature range to change our calculated value significantly. See Supporting Information (Table S1) for details.

To explore the thermal stability and reaction temperature of this compound, we performed DSC experiments on reacted and partially reacted samples of Hg_2_SiTe_4_, respectively. Our results suggest that the ternary compound is not stable above 500 °C and appears to begin volatilizing around 474 °C, as evidenced by a mass loss during our experiment. The reaction temperature is not determined with high confidence, but an endothermic event around 385 °C is observed and is close to our empirically derived hot press temperature of 400 °C, known to form our highest purity samples. Our experimental results and description are provided in Supporting Information (Figure S8).

### Defect Calculations

To investigate the dopability and role of native defects in Hg_2_SiTe_4_, we performed first-principles defect calculations. Given our prior work with Hg_2_GeTe_4_ in which mercury vacancy defects acted as electron “killer defects”, we chose to plot the defect formation energy of native defects in Hg_2_SiTe_4_ under the most Hg-rich chemical potential conditions.^6,17,21^ This way, we maximize the dopability window for *n*-type doping, as higher mobility is generally predicted for *n* vs *p*-type materials in this ordered vacancy chalcogenide space.^6^ Figure 5 displays the native defect formation energies under the most Hg-rich/Te-poor chemical potential conditions (bottom row of Table 2). First off, we note that the calculated HSE band gap is 1.2 eV, approximately double that of Hg_2_GeTe_4_.^17^ Several deep defects reside within the band gap, namely cation antisite defects ${\mathrm{Si}}_{\mathrm{Hg}}^{2+}$ and ${\mathrm{Hg}}_{\mathrm{Si}}^{2-}$ , as well as mercury vacancy $V_{\mathrm{Hg}}^{2-}$ defects. The equilibrium Fermi level is pinned within the band gap due to defect compensation, rendering doping difficult. We note that the measured Seebeck coefficient exhibits a pronounced temperature dependence (Figure 3c), which might appear to contradict our claim that the Fermi level is pinned in this chemical system. However, the defect formation energies are themselves a function of temperature, and a rising temperature may allow for some shift in *E*_*F*_ due to changing defect concentrations. Second, changes in scattering mechanisms, and in particular a positive energy dependence of the scattering time (as in ionized impurity scattering^22^) strongly affect *S*(*T*).

**Figure 5:**
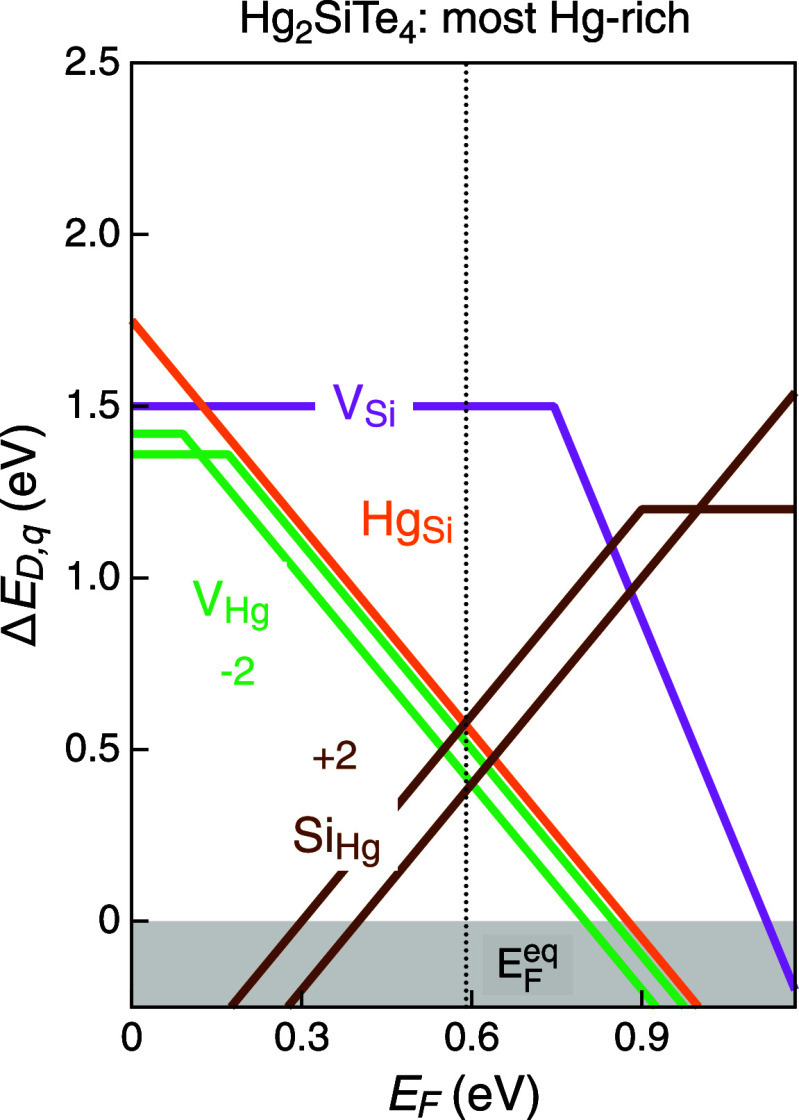
Defect calculations show that even under the most Hg-rich chemical potential conditions (bottom row of Table 2), ${\mathrm{Si}}_{\mathrm{Hg}}^{2+}$ and $V_{\mathrm{Hg}}^{2-}$ defects are low in energy at the equilibrium Fermi level.

As such, this semiconductor is unsuitable for thermoelectric applications as is, but it may be suitable for other electronic applications. The plethora of antisite and vacancy defects explains the low mobility (Figure 3) due to defect scattering. Finally, the abundance of native defects may also give rise to extremely low thermal conductivity due to strong point defect phonon scattering power between the heavy and light cations.^4^

## Conclusions

A new ordered vacancy chalcopyrite Hg_2_SiTe_4_ has been successfully synthesized and found to crystallize in the *I*4̅ space group and exhibit native *p*-type semiconductor behavior and ultralow thermal conductivity. The compound is similar both structurally and electronically to Hg_2_GeTe_4_. Defect calculations suggest that doping might be difficult due to low-energy native defects. Perhaps the most interesting discovery about this compound is that its formation requires densification prior to annealing to form the ternary, whereas Hg_2_GeTe_4_ (Ge analog) can be strictly ball-milled from the elements, and the resulting powder annealed to form a high-purity ternary compound.^5,6^ This suggests that the synthesis reaction in Hg_2_SiTe_4_ is more limited by diffusion than in the Ge analog.

## Supplementary Material



## Notes

Additional pXRD and experiment photos, effective medium model of electronic and thermal transport, and DSC results (PDF)
