# Supplementary material for: Synthesis, Crystal Structure, and Transport in Ordered Vacancy Compound Hg2SiTe4
Source: Inorg Chem. 2026 Mar 30;65(14):7660–5. doi: 10.1021/acs.inorgchem.5c05605 (PMC13080983; doi:10.1021/acs.inorgchem.5c05605)
Supplement: Supplementary file 1 [file ic5c05605_si_001.pdf]

— Supporting Information —

Synthesis, crystal structure, and transport in ordered vacancy compound Hg_2SiTe_4

Claire E. Porter,[*,†] Jiaxing Qu,[‡] Philip Yox,[¶] Terra Berriodi,[†] Annalise E. Maughan,[¶] Elif Ertekin,[‡] and Eric S. Toberer[†]

†*Materials Science, Colorado School of Mines, Golden, CO, 80401, USA*

‡*Mechanical Science & Engineering, University of Illinois at Urbana-Champaign, Urbana, IL 61801, USA*

¶*Chemistry, Colorado School of Mines, Golden, CO, 80401, USA*

E-mail: c7porter@gmail.com

1. pXRD and Photos of Various Synthetic Techniques Attempted

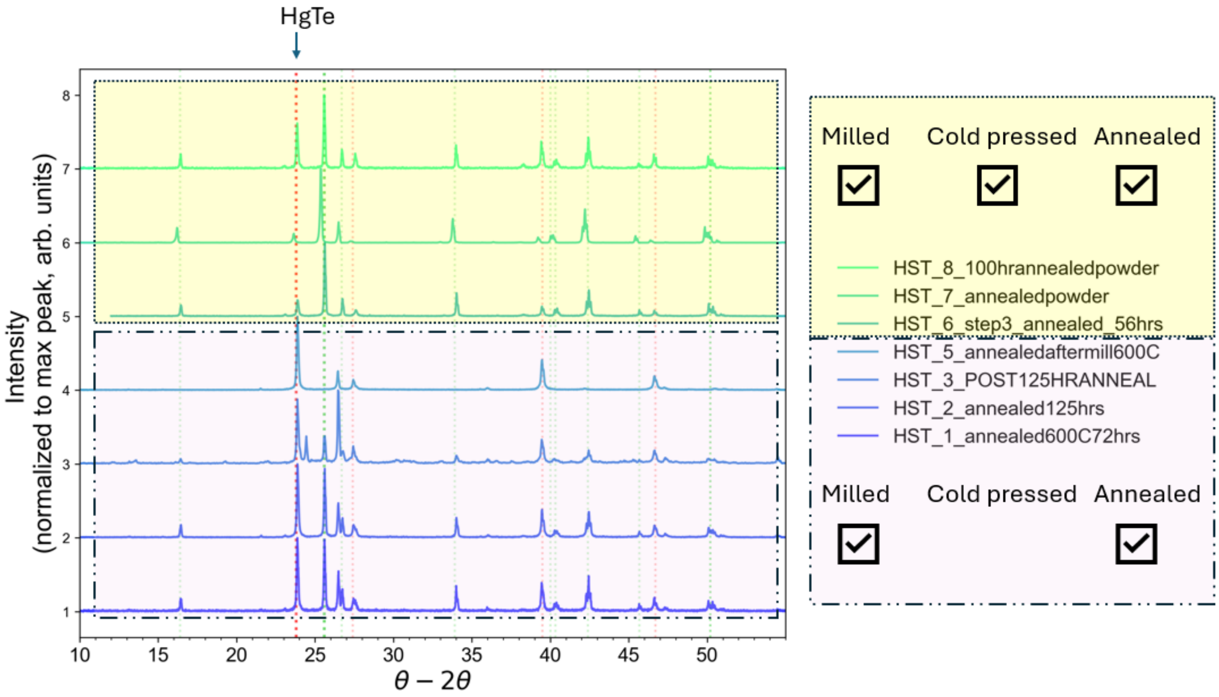

Figure S1: In gross, performing a cold press step after milling and before annealing results in successful formation of the ternary phase Hg_2SiTe_4, evidenced by a large peak at 2 $\theta = 25.6$ degrees. These experiments are highlighted on the top half of this plot. The bottom top half of this diffractogram shows that omitting the cold press step between the mill and the anneal can form some ternary phase Hg_2SiTe_4, but an unsatisfactorily high amount of HgTe remains unreacted.

Several different synthetic procedures were tested to optimize the synthesis of the Hg_2SiTe_4 compound after we confirmed its existence (Figure S1). The synthetic procedure that led to our initial discovery was as follows: (*i*) ball mill elemental constituents (Hg, Si, Te) in appropriate stoichimetric ratios for 60 minutes in an inert nitrogen-filled glovebox with oxygen levels under 1ppm, (*ii*) hand grind powder, load into evacuated fused silica ampoule for 24 hrs of annealing at 600°C, and (*iii*) press under vacuum at 40MPa and 400°C.

To determine the minimum synthesis required to form the ternary compound, we tested several synthetic techniques.

First off, milling the constituent elements in the appropriate stoichiometric ratios does not produce the ternary; largely HgTe is formed (FigureS2).

Melting binary (Si_2Te_3 and HgTe) constituents with the appropriate amount of Te to form stoichiometric Hg_2SiTe_4 at 1000°C did not produce satisfactory results. The quenched ampoule was blackened and extracting the material from the walls of the quartz ampoule was too difficult to render this method practical.

We also tried reacting the binaries with excess tellurium to form Hg_2SiTe_4, avoiding temperatures above

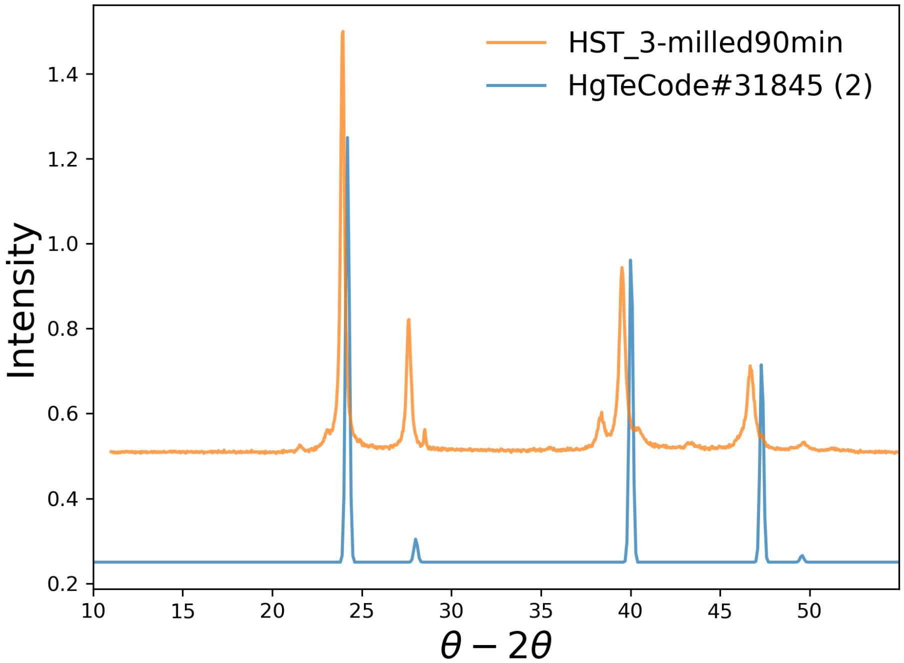

Figure S2: 90 minutes of milling stoichiometric amounts of Hg, Si, and Te together does not make the ternary.

600°C. The ampoule contained lovely red deposits, and when cracked open in air we smelled a pungent unpleasant odor. Both of these visual and olfactory cues suggest unreacted Si_2Te_3.

Once we realized that pellets of Hg_2SiTe_4 were fairly high purity ternary phase, but our milled/annealed powder was largely HgTe (evidenced from XRD, see Figure S4), we tried minutes to mix the elementsvia ball milling, then separate the hot press into a cold press, followed by a pressureless anneal. XRD was performed at each step to understand the impact on the reaction.

We performed XRD after the cold press to see if the press was sufficient to form the ternary, and it was not (middle trace in Figure S5). Therefore, the application of pressure (without heat), followed by a pressure-less anneal at 400°C (under ambient pressure, evacuated ampoule), is the minimum synthesis to achieve good results. If pressure is accompanied by heat (i.e. hot pressing), this will also create the ternary compound. However, heat alone is insufficient to react the elements to form Hg_2SiTe_4.

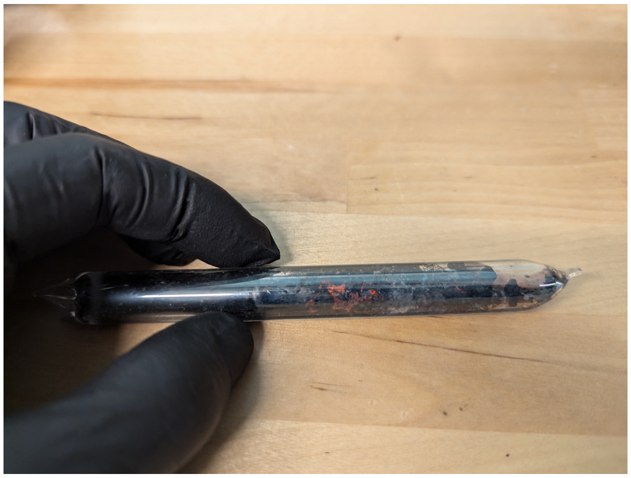

Figure S3: Red precipitates of Si_2Te_3 suggest incomplete reaction to form the ternary compound.

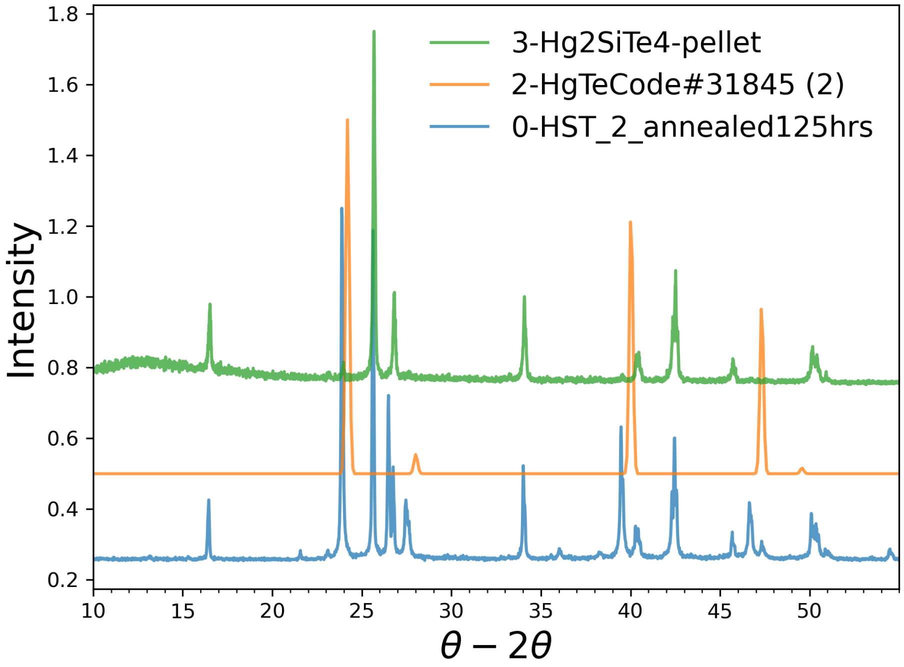

Figure S4: One of our original attempts (blue, buttom trace) that despite a 125 hr anneal at 600°C, a very high amount of unreacted HgTe remains.

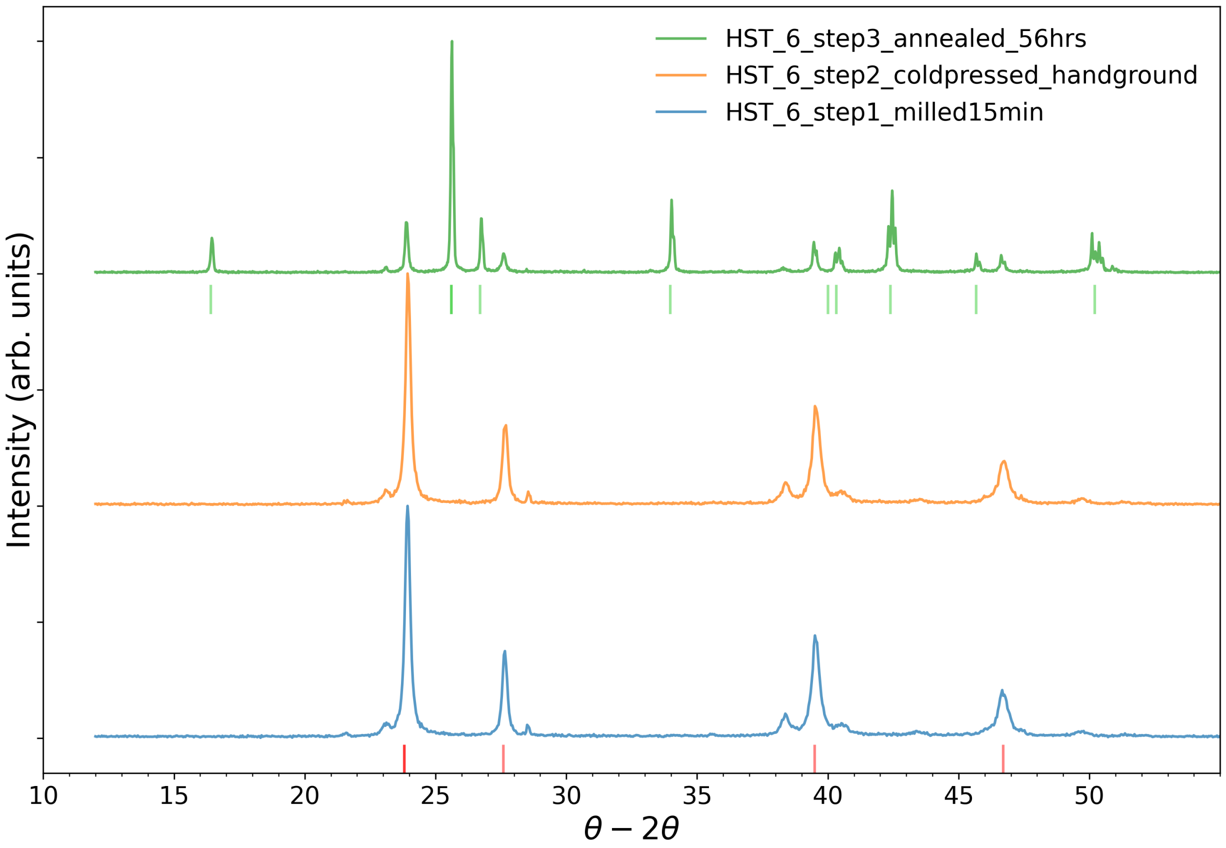

Figure S5: While the cold press is necessary to form the ternary phase, it is not sufficient. A 56 hr anneal at 600°C post-cold press results in decent phase purity Hg_2SiTe_4.

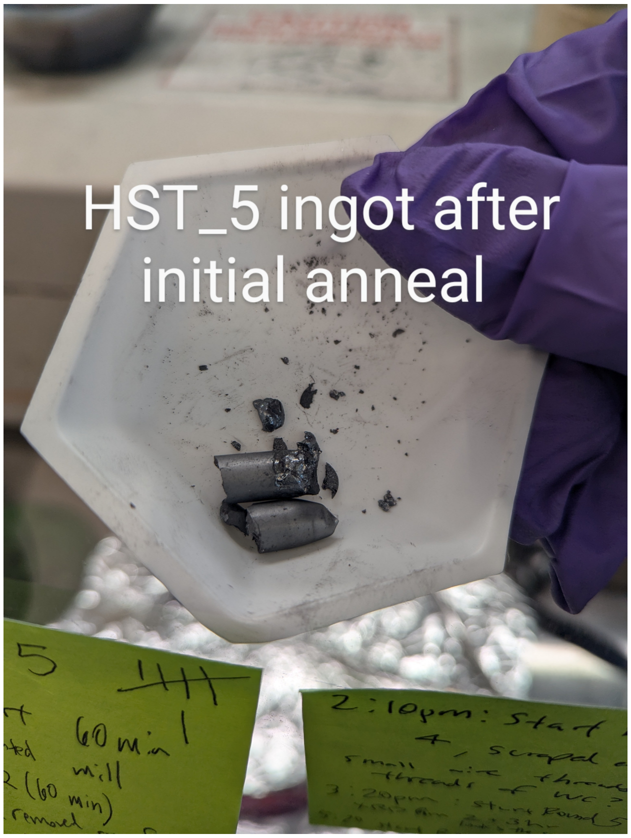

Figure S6: Hg_2SiTe_4 adopts a dark grey, mildly shiny hue.

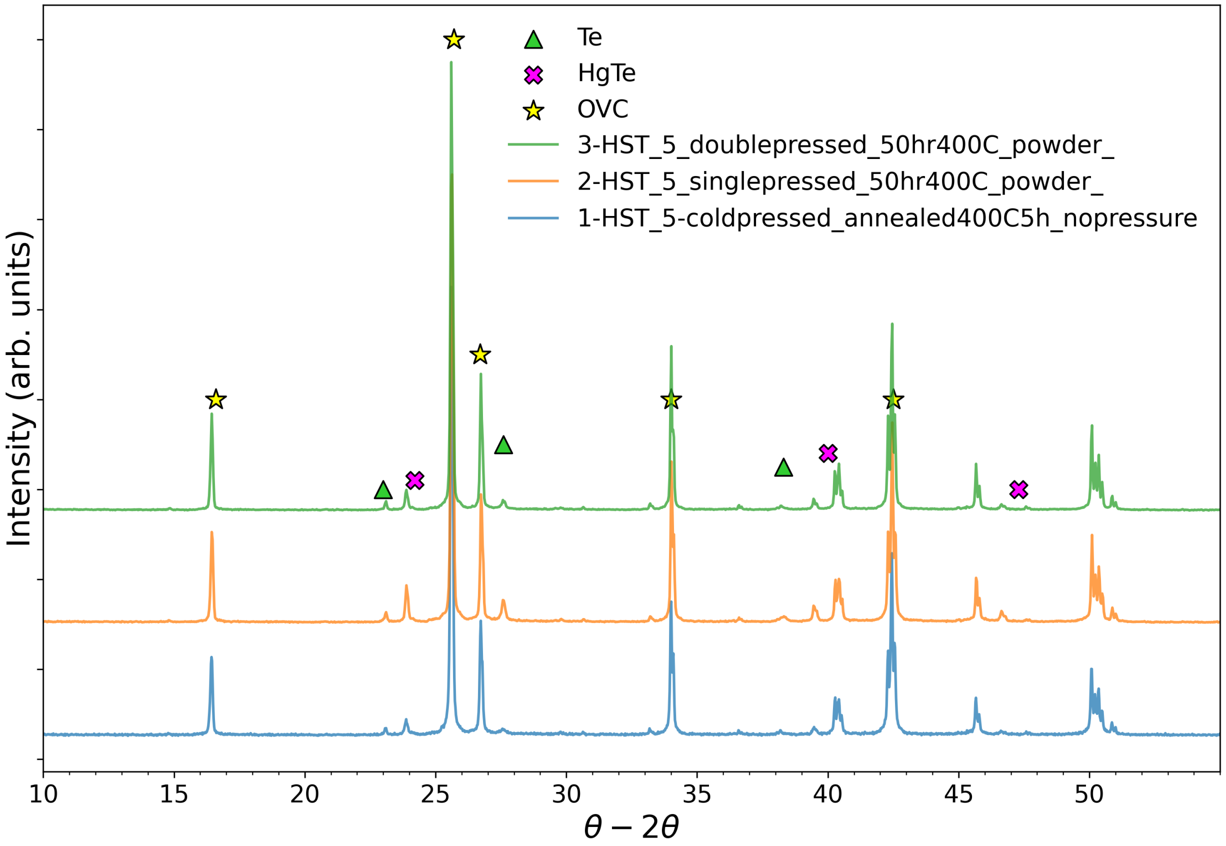

Figure S7: The top trace in this figure corresponds to powder that was cold pressed, annealed for 5 hrs at 400°C, hand ground, and then cold pressed again. We see a reduction in the amount of impurity phase HgTe present as a result of this re-grind/re-anneal.

2. Effective Medium Modeling of Thermal Conductivity & Transport Properties

We assumed a two phase mixture of 90 vol% Hg_2SiTe_4 and 10 vol % impurity phase (considered Si and HgTe separately) at room temperature and coded equations (1), (2), and (5) from Ref. [1] into Python to calculate the transport values for pure Hg_2SiTe_4. Code freely available upon request from corresponding author.

The effective (measured) values of resistivity, κ, and S used in the model are 3848 mΩcm, 0.69 W/m K, and 300 μV/K. All transport values for HgTe at room temperature were found in Ref. [2] Thermal and electrical conductivity values for silicon were found in Ref.,[3] and Seebeck coefficient data from Ref. [4]

Table S1: Calculated room temperature transport properties of pure Hg_2SiTe_4 considering the impact of HgTe or Si impurity phases using an effective medium theory model with 10 vol% of HgTe or silicon present.

Impurity Phase	Resistivity (mΩ cm)	Thermal Conductivity (W m^{-1} K^{-1})	Seebeck Coefficient (μV K^{-1})
HgTe	5449	0.58	446
Silicon	3300	0.49	300

3. Differential Scanning Calorimetry for Hg_2SiTe_4

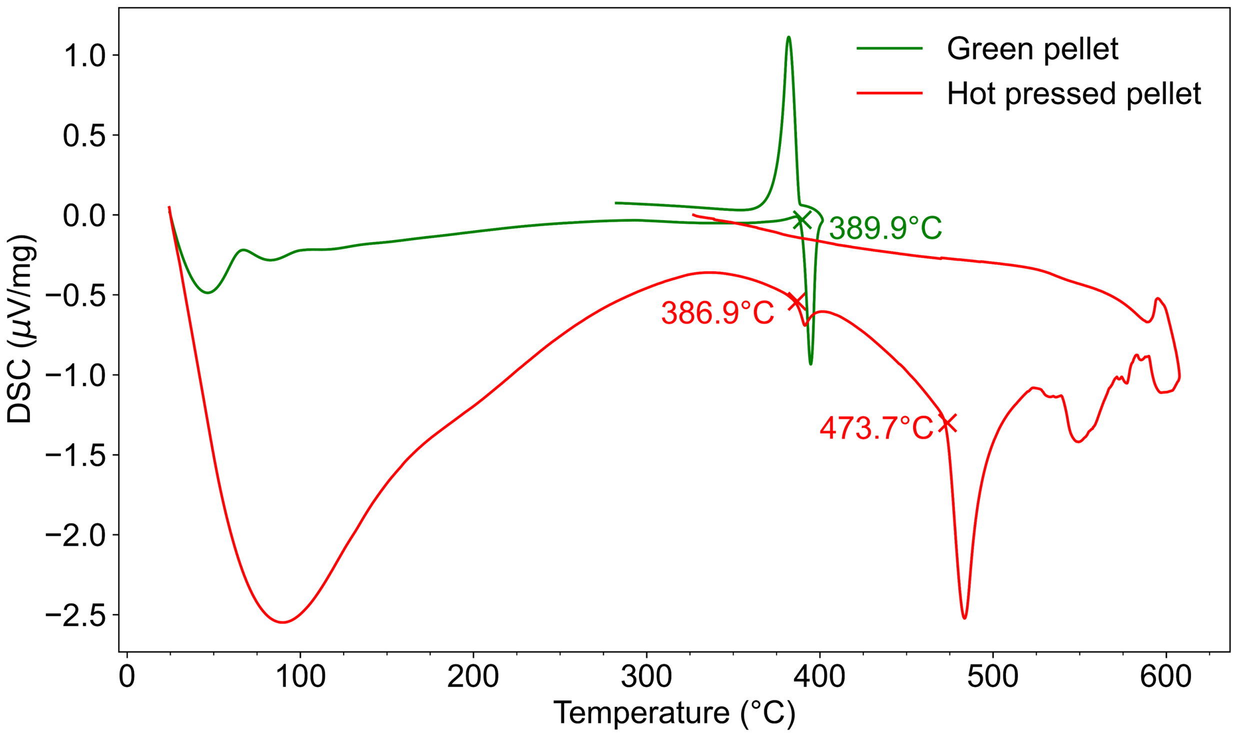

Figure S8: Differential scanning calorimetry (DSC) on a green body (top green curve) of mixed but unreacted precursors was performed from room temperature up to 400°C and the sample appears to have melted (endothermic data = negative DSC) and solidified upon cooling. The bottom trace (red) corresponds to our study to find the melting point. A hot pressed pellet was taken to 600°C and the melting point onset appears to be 474°C.

We conducted two DSC experiments: (1) we heated a hot pressed (400°C) pellet of Hg_2SiTe_4 to 600°C and recorded the heating and cooling curves, and (2) we subjected a green body of unreacted precursors to 400°C in an effort to determine the reaction temperature. Ultimately, our efforts were unsuccessful at pinpointing the exact reaction temperature, suggesting that the reaction might occur in multiple steps and/or involve several phase transitions between liquid to solid (e.g. Hg (liquid) to HgTe (solid)).

To find the melting temperature of Hg_2SiTe_4, we subjected a hot pressed (at 400°C) pellet of Hg_2SiTe_4 (over 80 wt% from XRD) to elevated temperature (600°C) and cooled the sample (bottom red trace in Figure S8b). The results from our DSC experiment suggest that the ternary begins melting around 474°C and is not stable at temperatures above 500°C. At the conclusion of this experiment, the sample was weighed and its mass was 40% less than the starting mass, suggesting the volatilization of a phase, possibly HgTe or Te.

We also observe from our DSC results that one step along the synthesis pathway involves a melting step at 386.9°C, very similar to the temperature at which a component contained in the green body melted (Figure S8b). The 390°C peak exhibited by both curves could be evidence of tellurium melting (eutectic of

HgTe-Te is 412°C). We do not observe a re-solidification at this temperature (bottom/red trace), which could be due to loss of the component as it evaporated (aberrant data between 500-600°C suggests volatilization of some elements or compounds).

To prepare the green body (top/green trace in Figure S8b), elements Hg, Si, and Te were weighed in appropriate stoichiometric ratios to form Hg_2SiTe_4 and were ball milled for 60 min in a N_2-containing glovebox. The resulted powder was scraped into a graphite die and pressed at room temperature for 5 minutes. The resulting cold pressed pellet was verified using XRD to not contain the ternary, but appears to be largely composed of HgTe and Te (Si might not appear due to its low mass). An endothermic event occurred at 389.9°C (Figure S8b), however we do not believe this to be the reaction temperature since an exothermic peak was observed upon cooling (solidification). If the sample reacted at 390°C, we would expect to see an endothermic peak unaccompanied by an exothermic peak.

References

(1) Vaney, J.-B.; Piarristeguy, A.; Ohorodniichuck, V.; Ferry, O.; Pradel, A.; Alleno, E.; Monnier, J.; Lopes, E. B.; Goncalves, A. P.; Delaizir, G.; others Effective medium theory based modeling of the thermoelectric properties of composites: comparison between predictions and experiments in the glass–crystal composite system Si 10 As 15 Te 75–Bi 0.4 Sb 1.6 Te 3. *Journal of Materials Chemistry C* **2015**, *3*, 11090–11098.

(2) Carlson, R. Electrical Properties of Mercury Telluride. *Physical Review* **1958**, *111*, 476.

(3) Shanks, H.; Maycock, P.; Sidles, P.; Danielson, G. Thermal conductivity of silicon from 300 to 1400 K. *Physical Review* **1963**, *130*, 1743.

(4) Geballe, T.; Hull, G. Seebeck effect in silicon. *Physical Review* **1955**, *98*, 940.
